# Comparative analysis of complete *Ilex* (Aquifoliaceae) chloroplast genomes: insights into evolutionary dynamics and phylogenetic relationships

**DOI:** 10.1186/s12864-022-08397-9

**Published:** 2022-03-14

**Authors:** Kewang Xu, Chenxue Lin, Shiou Yih Lee, Lingfeng Mao, Kaikai Meng

**Affiliations:** 1 Co-Innovation Center for Sustainable Forestry in Southern China, College of Biology and the Environment, Nanjing Forestry University, Nanjing, 510275 China; 2 Faculty of Health and Life Sciences, INTI International University, 71800 Nilai, Malaysia; 3 State Key Laboratory of Biocontrol and Guangdong Provincial Key Laboratory of Plant Resources, School of Life Sciences, Sun Yat-sen University, Guangzhou, China

**Keywords:** Aquifoliaceae, Chloroplast genome, Hypervariable regions, Phylogenomics, Relationship

## Abstract

**Background:**

*Ilex* (Aquifoliaceae) are of great horticultural importance throughout the world for their foliage and decorative berries, yet a dearth of genetic information has hampered our understanding of phylogenetic relationships and evolutionary history. Here, we compare chloroplast genomes from across *Ilex* and estimate phylogenetic relationships.

**Results:**

We sequenced the chloroplast genomes of seven *Ilex* species and compared them with 34 previously published *Ilex* plastomes. The length of the seven newly sequenced *Ilex* chloroplast genomes ranged from 157,182 bp to 158,009 bp, and contained a total of 118 genes, including 83 protein-coding, 31 rRNA, and four tRNA genes. GC content ranged from 37.6 to 37.69%. Comparative analysis showed shared genomic structures and gene rearrangements. Expansion and contraction of the inverted repeat regions at the LSC/IRa and IRa/SSC junctions were observed in 22 and 26 taxa, respectively; in contrast, the IRb boundary was largely invariant. A total of 2146 simple sequence repeats and 2843 large repeats were detected in the 41 *Ilex* plastomes. Additionally, six genes (*psaC*, *rbcL*, *trnQ*, *trnR*, *trnT*, and *ycf1*) and two intergenic spacer regions (*ndhC*-*trnV* and *petN*-*psbM*) were identified as hypervariable, and thus potentially useful for future phylogenetic studies and DNA barcoding. We recovered consistent phylogenetic relationships regardless of inference methodology or choice of loci. We recovered five distinct, major clades, which were inconsistent with traditional taxonomic systems.

**Conclusion:**

Our findings challenge traditional circumscriptions of the genus *Ilex* and provide new insights into the evolutionary history of this important clade. Furthermore, we detail hypervariable and repetitive regions that will be useful for future phylogenetic and population genetic studies.

**Supplementary Information:**

The online version contains supplementary material available at 10.1186/s12864-022-08397-9.

## Introduction

*Ilex* L., comprised of ca. 600 evergreen or deciduous tree and shrub species, is the only genus in the family Aquifoliaceae [1]. Members of the genus are mostly distributed in the tropics, with centers of species diversity located in tropical America and southeast Asia, but also extending into temperate regions [2, 3]. Most species of *Ilex*, including *I. cornuta* Lindl. et Paxt., *I. purpurea* Hassk., *I. paraguariensis* A. St.-Hil., and *I. rotunda* Thunb., have economic and horticultural value [4–8] and relatively broad ranges, although many species are narrowly endemic. To date, as many as 250 species of *Ilex* have been classified as endangered and placed on the International Union for Conservation of Nature (IUCN) red list [9].

In the past two decades, advances in sequencing technology and analytical methods have contributed to greater phylogenetic resolution within *Ilex*. Several loci from both the nuclear and plastid genomes, including *rbcL*, *trnL*-*trnF*, *atpB*-*rbcL*, nuclear ribosomal DNA internal transcribed spacers (nrITS), and chloroplast glutamine synthetase (*nepGS*), have been used to estimate phylogenetic relationships within the genus [10–17]. However, a broad and representative sample of *Ilex* species has not yet been achieved in any phylogenetic study; thus the phylogeny of *Ilex* remains largely unresolved [13, 16]. Furthermore, recent phylogenetic studies have revealed substantial incongruence between the nuclear and plastid topologies [10, 13–15]. Recent molecular phylogenies did not support traditional classifications of *Ilex* based on morphological features [18, 19]; however, these studies used only a few plastid or nuclear gene fragments and had generally poor resolution due to high conservation of plastid genes. At present, the phylogenetic relationships among lineages in genus *Ilex* remain uncertain, thus, further investigations are needed to reconstruct the evolutionary history of this clade.

Complete chloroplast genomes have been relatively more successful than short sequence fragments in resolving the relationships of many land plant clades at different taxonomic levels [20–22]. In general, land plant chloroplast genomes are relatively stable and contain four extremely evolutionarily conserved regions: a pair of inverted repeat regions (IRa and IRb), a large single-copy region (LSC), and a small single-copy region (SSC) [23]. At the same time, chloroplast genomes contain a large amount of phylogenetic information with a mutation rate sufficient for phylogenetic inference and species delimitation [24].

To date, the complete chloroplast genome sequences of a total of 34 *Ilex* species have been made available in the NCBI GenBank database (accessed on 1 August 2021), which accounts for only ca. 5.7% of the total species diversity. Here, we expand *Ilex* genetic resources by newly sequencing the chloroplast genomes of seven species: *I. dasyphylla*, *I. fukienensis*, *I. lohfauensis*, *I. venusta*, *I. viridis*, *I. yunnanensis*, and *I. zhejiangensis*. Three of which, *Ilex fukienensis*, *I. venusta*, and *I. zhejiangensis*, are known to have a very narrow distribution in China [15, 25], while the other four species are widely distributed in China and adjacent regions. We aimed to (i) investigate the structural and compositional variations of *Ilex* chloroplast genomes, (ii) identify highly variable regions useful for resolving interspecific relationships and species delimitation, and (iii) test the cyto-nuclear discordance by reconstructing high-resolution phylogenetic trees.

## Results

### Chloroplast genome structure of *Ilex*

**Fig. 1:**
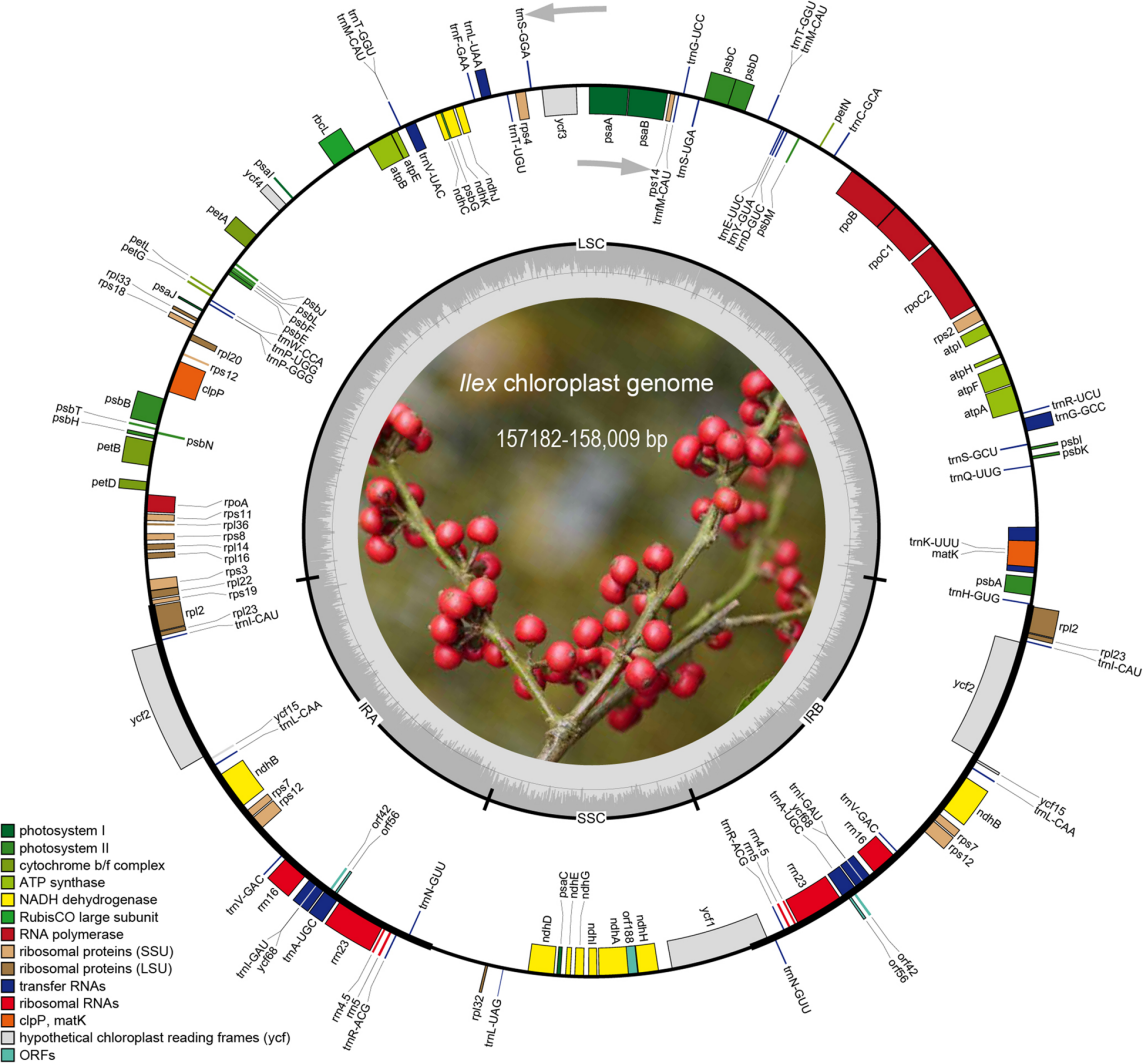
Gene circle maps of seven newly sequenced *Ilex* species. The colored bars indicate different functional groups. Thick lines of the large circle indicate the extent of the inverted repeat regions (IRa and IRb), which separate the genome into small (SSC) and large (LSC) single copy regions. Genes on the inside and outside of the large circle are transcribed clockwise and counterclockwise, respectively. The darker gray columns in the inner circle correspond to the GC content, the light gray to AT content

All seven newly sequenced *Ilex* chloroplast genomes possessed typical vascular plant quadripartite structure, which consisted of two single-copy regions (LSC and SSC) that were separated by a pair of inverted repeats (IRa and IRb) (Fig. 1). The length of the newly sequenced chloroplast genomes ranged from 157,182 bp (*I. zhejiangensis*) to 158,009 bp (*I. dasyphylla*). The length of the LSC regions ranged from 86,575 bp (*I. zhejiangensis*) to 87,389 bp (*I. yunnanensis*), SSC regions ranged from 18,228 bp (*I. yunnanensis*) to 18,447 bp (*I. lohfauensis*), and IR regions ranged from 26,065 bp (*I. viridis*) to 26,108 bp (*I. yunnanensis*) (Table 1). The GC content ranged from 37.62% (*I. dasyphylla*) to 37.69% (*I. zhejiangensis*) (Table 1). All newly assembled plastomes contained 117 genes, including 82 protein-coding, 31 tRNA, and four rRNA genes, except for *I. dasyphylla*, which had 83 protein-coding genes (Tables 2 and 3). All chloroplast genomes had the same gene order and arrangement.

**Table 1:** Summary of complete chloroplast genomes of *Ilex* species included in the present study. PCG indicates protein-coding gene

Taxon	Accession number	Gene number				Length (bp)				GC(%)	AT(%)
		PCG	tRNA	rRNA	Full	Plastome	LSC	IRA/IRB	SSC		
*Ilex asprella*	NC_045274	94	37	8	139	157,856	87,265	26,075	18,441	37.62	62.38
*I. asprella* var. *tapuensis*	MT767004	87	37	8	132	157,671	87,161	26,065	18,380	37.65	62.35
*I. championii*	MT764248	87	37	8	132	157,468	86,878	26,074	18,442	37.64	62.36
*I. chapaensis*	MT764251	87	37	8	132	157,665	87,155	26,065	18,380	37.65	62.35
*I. cinerea*	MT764247	87	37	8	132	157,215	86,601	26,094	18,426	37.69	62.31
*I. cornuta*	MT764252	87	37	8	132	157,216	86,607	26,091	18,427	37.69	62.31
*I. crenata*	MW528027	88	38	8	134	157,988	87,414	26,076	18,422	37.65	62.35
*I. dabieshanensis*	MT435529	90	37	8	135	157,218	86,723	26,034	18,427	37.69	62.31
*I. dasyphylla*	This study	92	40	8	140	158,009	87,388	26,105	18,411	37.62	62.38
*I. dasyphylla*	MT764253	87	37	8	132	158,009	87,388	26,105	18,411	37.62	62.38
*I. delavayi*	KX426470	95	40	8	143	157,671	87,077	26,078	18,438	37.65	62.35
*I. dumosa*	KP016927	86	37	8	131	157,732	87,033	26,087	18,415	37.62	62.27
*I. ficoidea*	MT764243	87	37	8	132	157,536	86,922	26,094	18,426	37.64	62.36
*I. fukienensis*	This study	91	40	8	139	157,474	86,886	26,074	18,440	37.64	62.36
*I. graciliflora*	MT764249	87	37	8	132	157,119	86,506	26,093	18,427	37.61	62.39
*I. hanceana*	MT764246	87	37	8	132	157,478	86,889	26,074	18,441	37.63	62.37
*I. integra*	NC_044417	86	37	8	131	157,548	86,936	26,093	18,426	37.64	62.36
*I. intermedia*	MT471320	89	37	8	134	157,577	87,083	26,034	18,426	37.63	62.37
*I. kwangtungensis*	MT764241	87	37	8	132	158,020	87,400	26,104	18,412	37.62	62.38
*I. lancilimba*	MT767005	87	37	8	132	157,998	87,382	26,105	18,406	37.62	62.38
*I. latifolia*	NC_047291	95	40	8	143	157,601	87,020	26,077	18,427	37.63	62.37
*I. lohfauensis*	This study	91	40	8	139	157,464	86,875	26,071	18,447	37.64	62.36
*I. lohfauensis*	MT764240	87	37	8	132	157,470	86,873	26,078	18,441	37.64	62.36
*I. memecylifolia*	MT764250	87	37	8	132	157,842	87,249	26,076	18,441	37.62	62.38
*I. micrococca*	MN830251	89	37	8	134	157,782	87,200	26,074	18,434	37.64	62.36
*I. paraguariensis*	NC_031207	86	37	8	131	157,614	87,154	26,076	18,308	37.63	62.35
*I. polyneura*	KX426468	95	40	8	143	157,621	87,140	26,061, 25,980	18,434	37.60	62.40
*I. pubescens*	KX426467	95	40	8	143	157,741	87,143	26,081	18,436	37.61	62.39
*I. purpurea*	MT471318	89	37	8	134	157,885	87,289	26,104	18,388	37.62	62.38
*I. rotunda*	MW292559	88	37	8	133	157,743	87,069	26,121	18,432	37.62	62.38
*I.* sp.	KX426469	95	40	8	143	157,611	87,137	26,020	18,434	37.62	62.38
*I. suaveolens*	MN830249	89	37	8	134	157,857	87,255	26,102	18,398	37.65	62.35
*I. szechwanensis*	KX426466	95	40	8	143	157,822	87,281	26,053	18,435	37.65	62.35
*I. triflora*	MT764242	87	36	8	131	157,706	87,183	26,065	18,393	37.67	62.33
*I. venusta*	This study	91	40	8	139	157,860	87,290	26,079	18,412	37.66	62.34
*I. viridis*	This study	91	40	8	139	157,673	87,150	26,065	18,393	37.68	62.32
*I. viridis*	MN830250	89	37	8	134	157,701	87,177	26,065	18,394	37.67	62.33
*I. vomitoria*	MT471319	90	36	8	134	157,328	86,920	26,005	18,398	37.66	62.34
*I. wilsonii*	KX426471	95	40	8	143	157,918	87,341	26,073	18,431	37.62	62.38
*I. yunnanensis*	This study	91	40	8	139	157,833	87,389	26,108	18,228	37.65	62.35
*I. zhejiangensis*	This study	91	40	8	139	157,182	86,575	26,092	18,423	37.69	62.31

**Table 2:** List of annotated genes in the chloroplast genomes of the *Ilex* species

Function of Genes	Group of Genes	Gene Name
Protein synthesis and DNA-replication	Transfer RNAs	*trnC-GCA*, *trnD-GUC*, *trnE-UUC*, *trnF-GAA*, *trnfM-CAU*, *trnG-GCC*^a^, *trnG-UCC*, *trnH-GUG*, *trnK-UUU*^a^*,*, *trnL-UAA*^a^*,*, *trnM-CAU*, *trnQ-UUG*, *trnP-GGG*, *trnP-UGG*, *trnR-UCU*, *trnS-GCU*, *trnS-GGA*, *trnS-UGA*, *trnT-GGU* (× 2), *trnT-UGU*, *trnV-UAC*^a^*,*, *trnW-CCA*, *trnY-GUA*, *trnA-UGC*^a^*,* (× 2), *trnI-CAU* (× 2), *trnI-GAU*^a^*,* (× 2), *trnL-CAA* (× 2), *trnL-UAG*, *trnN-GUU* (× 2), *trnR-ACG* (× 2), *trnV-GAC* (× 2), *trnM-CAU*
	Ribosomal RNAs	*rrn4.5* (× 2), *rrn5* (× 2), *rrn16* (× 2), *rrn23* (× 2)
	Ribosomal protein large subunit	*rpl33*, *rpl20*, *rpl36*, *rpl14*, *rpl16*, *rpl22*, *rpl32*, *rpl2*^a^*,* (× 2), *rpl23* (× 2)
	Ribosomal protein small subunit	*rps2*, *rps14*, *rps4*, *rps18*, *rps11*, *rps8*, *rps3*, *rps19*, *rps12*^a^*,* (× 2), *rps7* (× 2)
	Subunits of RNA polymerase	*rpoA*, *rpoB*, *rpoC1*^a^*,*, *rpoC2*
Photosynthesis	photosystem I	*psaA*, *psaB*, *psaC*, *psaI*, *psaJ*
	Photosystem II	*psbA*, *psbB*, *psbC*, *psbD*, *psbE*, *psbF*, *psbG*, *psbH*, *psbI*, *psbJ*, *psbK*, *psbL*, *psbM*, *psbN*, *psbT*, *lhbA*
	ATP synthase	*atpA*, *atpB*, *atpE*, *atpF*^a^*,*, *atpH*, *atpI*
	Large subunit Rubisco	*rbcL*
	Cythochrome b/f complex	*petA*, *petB*^a^*,*, *petD*, *petG*, *petL*, *petN*
	NADH-dehydrogenase	*ndhA*^a^*,*, *ndhB*^a^*,* (× 2), *ndhC*, *ndhD*, *ndhE*, *ndhG*, *ndhH*, *ndhI*, *ndhJ*, *ndhK*
Other genes	Translation initiation factor	*infA*
	Cytochrome c biogenesis	*ccsA*
	ATP-dependent protease	*clpP*^b^
	Maturase	*matK*
	Inner membrane protein	*cemA*
	Acetyl-CoA carboxylase	*accD*
Genes of unknown function	Conserved hypothetical gene	*orf42* (× 2), *orf56* (× 2), *orf188*, *ycf3*^b^, *ycf4*, *ycf1*, *ycf2* (× 2), *ycf15* (× 2), *ycf68* (× 2)

Note: (× 2) indicates the number of repeat units is 2; ^a^Gene contains a single intron; ^b^Gene contains two introns

**Table 3:** Genes with introns in the chloroplast genome of *Ilex* species

Gene	Location	Exon I(bp)	Intron I (bp)	Exon II (bp)	Intron II (bp)	Exon III (bp)
*rpl2*	IRa + IRb	393	661	435		
*rps12*	LSC + IRs	114	543	232		26
*clpP*	LSC	69	819	291	602	78
*atpF*	LSC	159	681	408		
*rpoC1*	LSC	456	756	1635		
*ndhA*	SSC	552	1140	540		
*ndhB*	IRA	777	679	756		
*petB*	LSC	6	745	657		
*trnA-UGC*	IRa + IRb	38	807	35		
*trnI-GAU*	IRa + IRb	42	934	35		
*trnL-UAA*	IRa + IRb	37	490	50		
*trnV-UAC*	IRa + IRb	39	579	37		
*trnG-GCC*	LSC	23	703	48		
*trnK-UUU*	LSC	37	2562	35		
*ycf3*	LSC	126	727	228	749	153

### Comparative analysis of genomic divergence and genome rearrangement

**Fig. 2:**
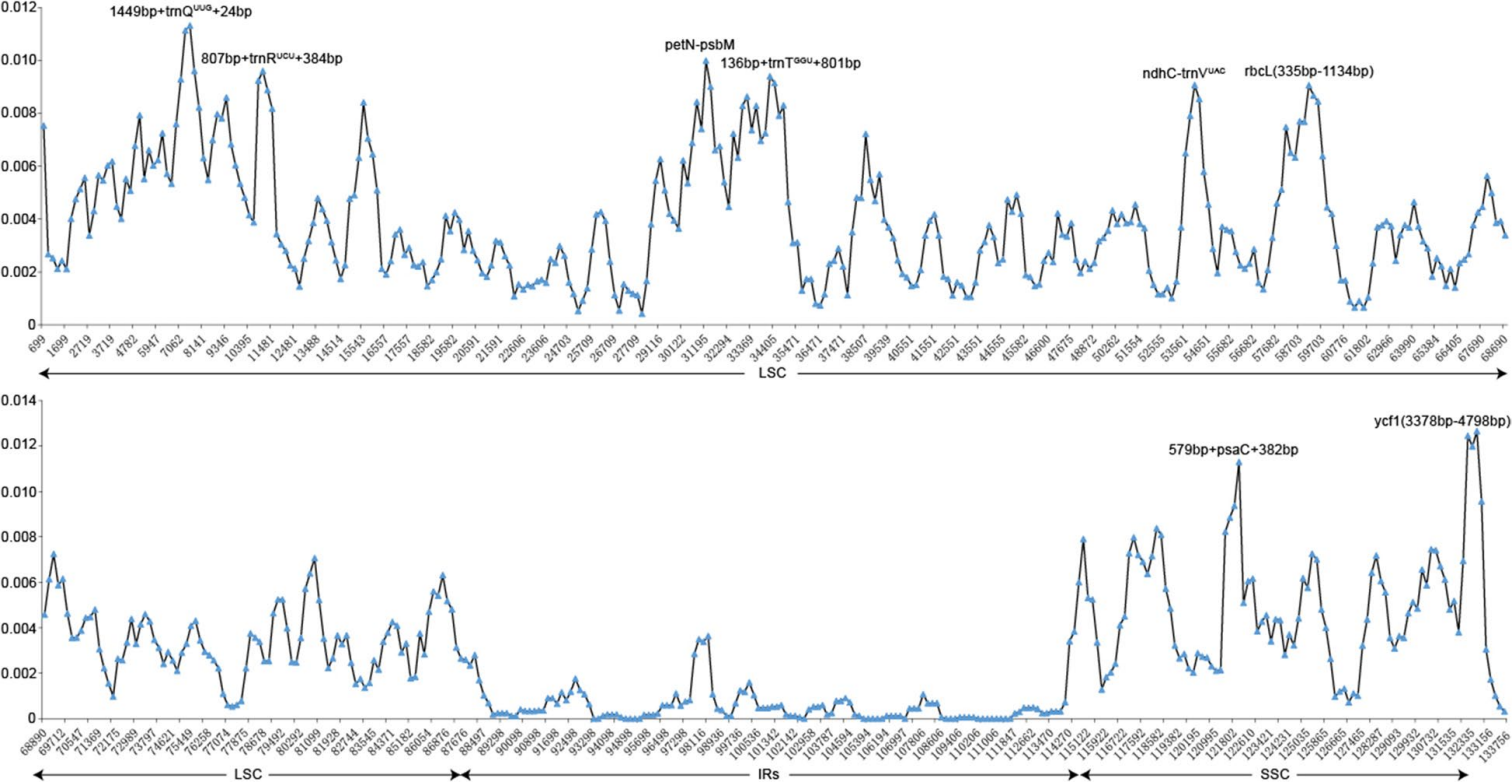
Sliding-window analysis showing the nucleotide diversity (Pi) values of the aligned *Ilex* chloroplast genomes

The diversity of nucleotide variability (Pi) for the seven newly assembled plastomes, combined with 34 plastomes obtained from GenBank, ranged from 0.000 to 0.01266, with an average of 0.00286. Based on the cutoff value of Pi ≥0.009, eight highly variable regions (807 bp + *trnR*^UCU^ + 384 bp, 579 bp + *psaC* + 382 bp, *ycf1* (3378 bp–4798 bp), 136 bp + *trnT*^GGU^ + 801 bp, *rbcL* (335 bp–1134 bp), *ndhC*-*trnV*^UAC^, 1449 bp + *trnQ*^UUG^ + 24 bp, and *petN*-*psbM*) were identified; six of which (*rbcL*, *trnQ*, *trnR, trnT*, *ndhC*-*trnV*, and *petN*-*psbM*) were located in the LSC region, while two (*psaC* and *ycf*1) were from the SSC region (Fig. 2, Additional file 1: Table S1). The Pi value of the eight hypervariable loci ranged from 0.00754 (807 bp + *trnR*^UCU^ + 384 bp) to 0.00955 (*petN-psbM*) (Table 4). At least four distinct gaps were observed in the chloroplast genome alignment, all located in the LSC region (Additional file 2: Fig. S1) within intergenic spacer regions, including *cemA*-*ycf4*, *petA*-*psbJ*, *rpoB*-*trnC*, and *trnL*-*trnT*. Four species (*I. championii*, *I. fukienensis*, *I. hanceana*, and *I. lohfauensis*) had a gap at the *rpoB-trnC* region, while three species (*I. polyneura*, *I. pubescens*, and *I. rotunda*) had a gap at the *petA-psbJ* region. Species that contained gaps at the *cemA*-*ycf4* region also contained gaps at the *trnL*-*trnT* region, which included *I. cinerea*, *I. cornuta*, *I. dabieshanensis*, *I. ficoidea*, *I. graciliflora*, *I. intermedia*, *I. latifolia*, *I. zhejiangensis*, and *Ilex* sp. However, two species, *I. delavayi*, and *I. integra* only had one of these gaps, which was at the *cemA-ycf4* region. Upon manual checking, these variations represented indels, ranging from about 210 bp (*petA-psbJ*) to 379 bp (*rpoB-trnC*) in length. Genome synteny of the 41 chloroplast genomes revealed no large gene rearrangement events (Additional file 2: Fig. S2). In addition, a total of 2947 polymorphic, 1630 singleton variable, and 1317 parsimony-informative sites were detected in the 41 chloroplast genome sequences.

**Table 4:** Variable site analyses in the chloroplast genomes of *Ilex* species

Region	Total number of sites	Polymorphic sites	Singleton variable sites	Parsimony informative sites	Nucleotide diversity
LSC	88,362	2182	1200	982	0.00384
IRa	26,162	94	57	37	0.00055
SSC	18,460	582	319	263	0.00498
IRb	26,167	89	54	35	0.00050
Plastome	159,151	2947	1630	1317	0.00286

### Expansion and contraction of the IR regions

**Fig. 3:**
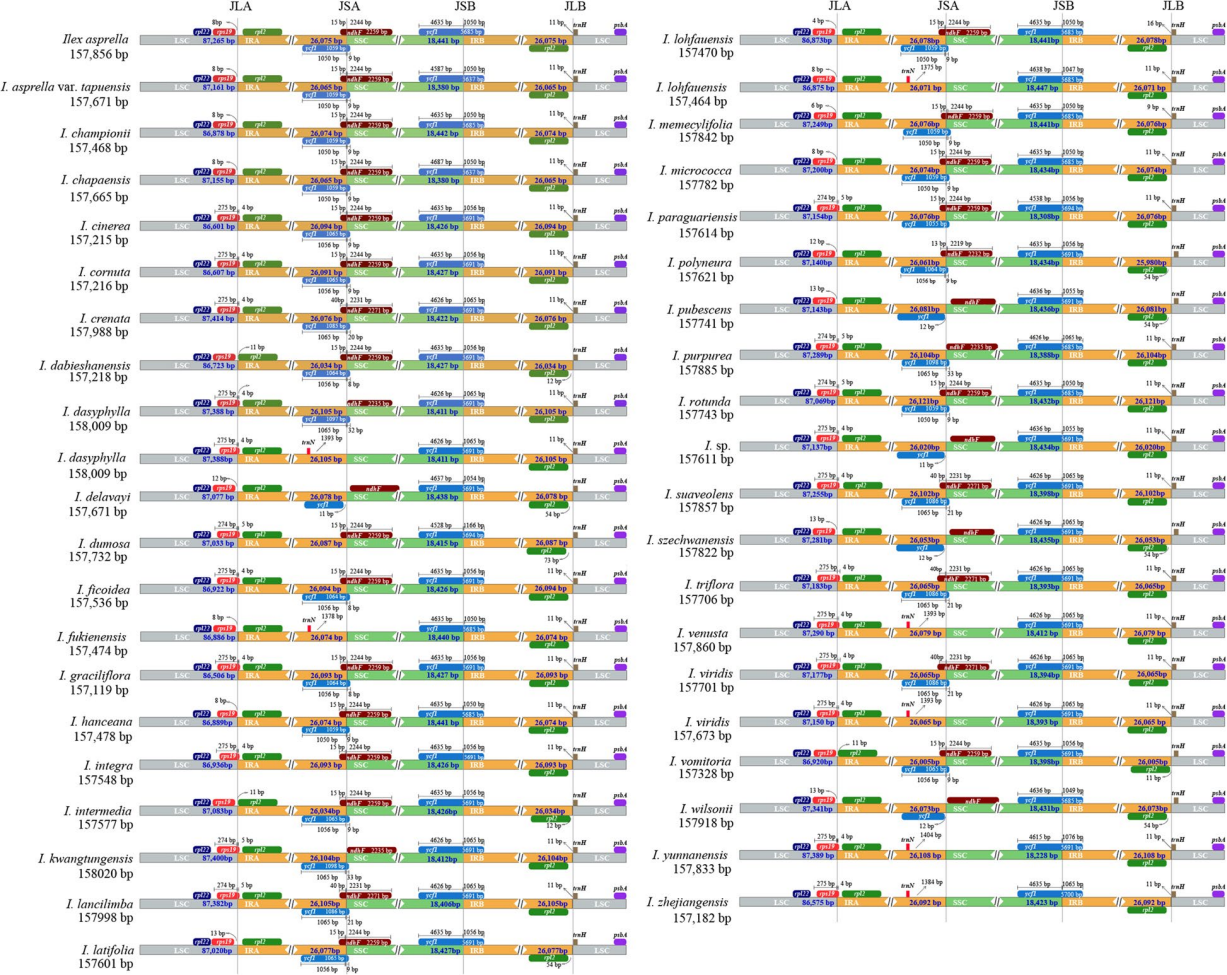
Comparison of the SC/IR junctions among the 41 *Ilex* chloroplast genomes. JLA, LSC/IRa boundary; JSA, SSC/IRa boundary; JSB, SSC/IRb boundary; JLB, LSC/IRb boundary

Comparative sequence analysis of the *Ilex* species showed that chloroplast genome structure and the number and sequence of genes were highly conserved. However, some structure and size variations at the IR boundaries were detected. The lengths of IRs among all *Ilex* species analyzed were relatively consistent: *I. vomitoria* had the shortest (26,005 bp), while *I. rotunda* had the longest (26,121 bp). About half (22/41) of the *Ilex* plastomes had LSC/IRa junctions located in *rps19*, with 4 to 5 bp crossing into the IRa region, which indicated an expansion of the IR in these species (Fig. 3). The majority of IRa/SSC junctions were located adjacent to *ycf1* and *ndhF*, and overlap of 22 to 61 bp between *ndhF* and *ycf1* was detected in 26 species. However, in *I. dasyphylla*, *I. fukienensis*, *I. lohfauensis*, *I. venusta*, *I. viridis*, *I. yunnanensis*, and *I. zhejiangensis*, *ndhF* and *ycf1* were absent from the IRa/SSC junction. In all analyzed *Ilex* chloroplast genomes, the SSC/IRb junction was located in *ycf1*, with an extension into the IRb region ranging from 1047 bp (*I. lohfauensis*) to 1166 bp (*I. dumosa*) (Fig. 3).

### SSR polymorphisms and long repeat sequence analysis

**Fig. 4:**
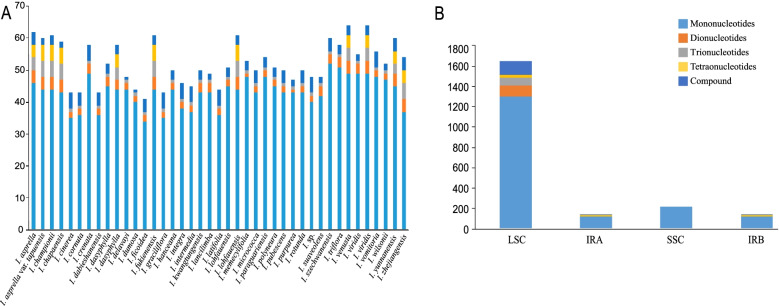
Analysis of simple sequence repeats (SSR) in the 41 chloroplast genomes of *Ilex* species. **A** Number of different SSR types detected in the 41 genomes; **B** Number of different SSR types in LSC, SSC and IR regions

A total of 2146 simple sequence repeats (SSRs) were detected among the 41 *Ilex* chloroplast genomes, ranging from 10 to 168 bp (Fig. 4, Additional file 1: Table S2). Mononucleotide repeats were most abundant (1771), while tetranucleotide repeats were rarest (49). The number of di-, trinucleotide, and compound repeats were 109, 79, and 138, respectively. Of the mononucleotide repeats, A/T repeats were most frequent (1769), while C/G repeats were only detected from two taxa (*I. asprella* var. *tapuensis* and *I. micrococca*). Dinucleotide repeats were represented by only the AT/TA motif; while tri- and tetranucleotides contained motifs AAT/ATT, CAG/CTG, and TTC/GAA, as well as AAAG/CTTT, ATAA/TTAT, ATTT/AAAT, TATT/AATA, and TCTT/AAGA repeats, respectively. Most SSRs were located in LSC regions (1649), followed by IR (275), and SSC (222) regions.

**Fig. 5:**
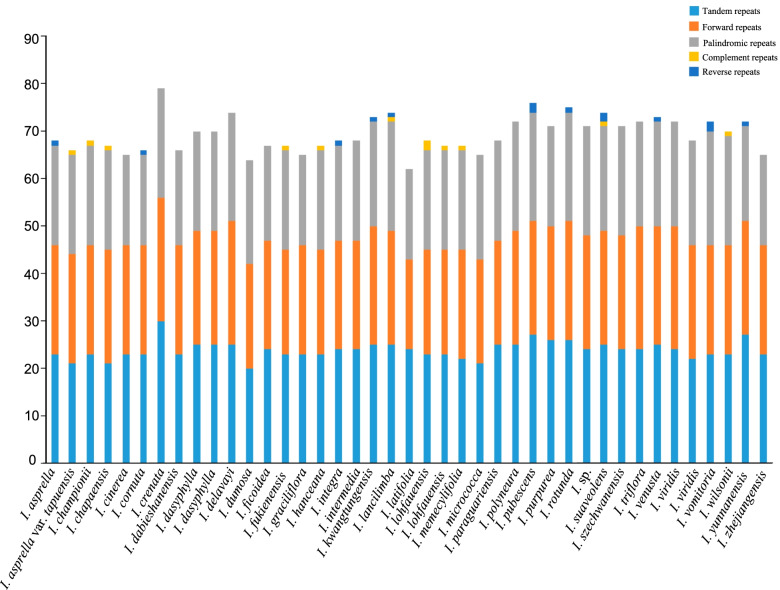
Analysis of long repeats in 41 chloroplast genomes of *Ilex* showing the number of complementary, forward, palindromic, reverse, and tandem long repeats

We detected a total of 2843 large repeats between the 41 species (Fig. 5, Additional file 1: Table S3); *I. crenata* had the highest (79), while *I. latifolia* the fewest (62), large repeats. All species involved had forward, palindromic, and tandem repeats, but only 11 had complementary and/or reverse repeats.

### Phylogenomic analyses

**Fig. 6:**
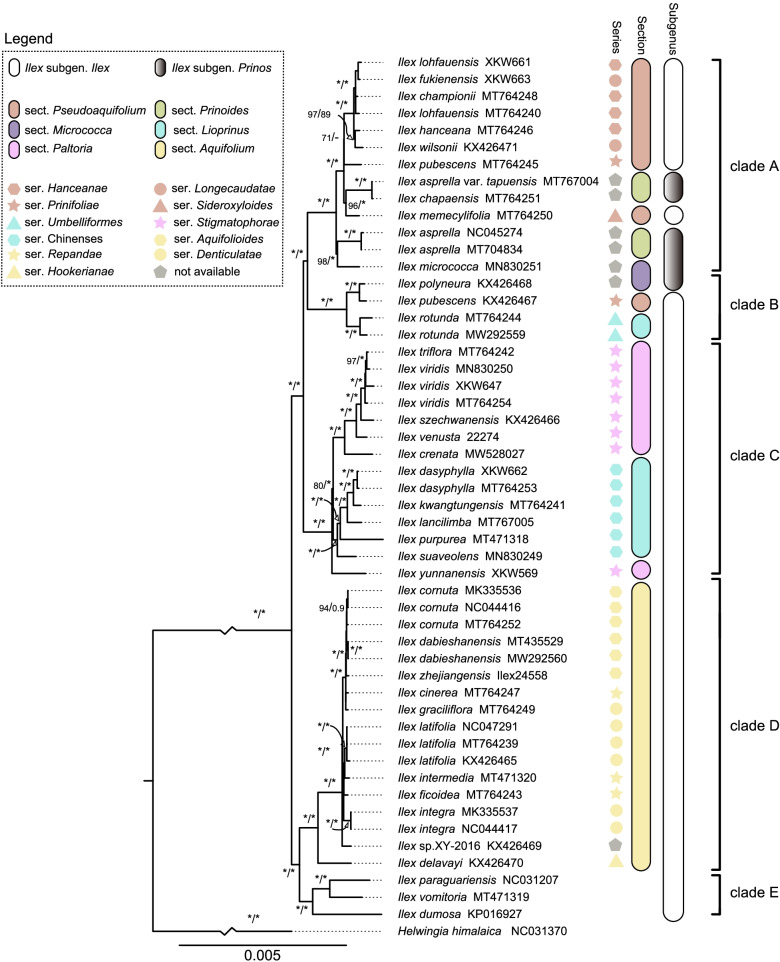
Phylogenetic trees inferred from maximum likelihood (ML) and Bayesian inference (BI) analyses based on the complete chloroplast genomes. Numbers near the nodes are ML bootstrap support values (BS, left of the slashes) and Bayesian posterior probabilities (PP, right of the slashes). 100% BS or 1.00 PP are indicated by asterisks. Incongruences between the BI and ML trees are indicated by dashes. Hu’s classification is illustrated by color graphic pattern. Recognized groups (major clades) were also marked by the right-hand black bar

We reconstructed phylogenetic relationships from 52 complete chloroplast genomes and 75 protein-coding genes using both maximum likelihood (ML) and Bayesian inference (BI) methods, and used the closely related species *Helwingia himalaica* (NC031370) as an outgroup [26]. The total alignment lengths of the complete plastome and the protein-coding gene matrices were 157,836 bp and 68,601 bp, respectively. The complete plastome matrix contained 8869 variable and 1735 parsimony informative sites, while the protein-coding gene matrix contained 2247 and 458 variable and parsimony informative sites, respectively. The backbones of trees constructed using ML and BI methods were almost identical for each sequence matrix and supported the monophyly of *Ilex* (Fig. 6; ML BS: 100%; BI PP: 1.00); thus, we present only the ML tree here, with posterior probability (PP) values shown (Fig. 6, Additional file 2: Fig. S3).

Based on our phylogenetic analyses, and with consideration of macro-morphological and distribution information, we recognize five highly supported clades within *Ilex* (clades A–E) that were well resolved (Fig. 6; ML BS: 100%; BI PP: 1.00). Clade A comprises one species (*I. micrococca*) of sect. *Micrococca*, two species (*I. asprella* and *I. chapaensis*) and one variety (*I. asprella* var. *tapuensis*) of sect. *Prinoides*, and seven species (*I. championii*, *I. fukienensis*, *I. hanceana*, *I. lohfauensis*, *I. memecylifolia*, *I. pubescens*, and *I. wilsonii*) of sect. *Pseudoaquifolium*. Clade B is sister to clade A, and includes three species (*I. polyneura*, *I. pubescens*, and *I. rotunda*). Clade C contains five species (*I. dasyphylla, I. kwangtungensis*, *I. lancilimba*, *I. purpurea*, and *I. suaveolens*) from sect. *Lioprinus* and six species (*I. crenata*, *I. szechwanensis*, *I. triflora*, *I. venusta*, *Ilex viridis*, and *I. yunnanensis*) from sect. *Paltoria*. Clade D includes members from sect. *Aquifolium*, and is sister to Clade E, which only contains three species (*I. dumosa*, *I. paraguariensis*, and *I. vomitoria*). Only sect. *Aquifolium* was resolved as monophyletic, while the other five sections (*Lioprinus*, *Micrococca*, *Paltoria*, *Prinoides*, and *Pseudoaquifolium*) and six series (*Denticulatae*, *Hanceanae*, *Longecaudatae*, *Prinifoliae*, *Repandae*, and *Stigmatophorae*) were not. Interspecific relationships within each clade were generally well resolved with high support.

## Discussion

### Comparison *Ilex* chloroplast genomes

We found that *Ilex* possesses typical, quadripartite chloroplast genomes at sizes consistent with most land plants [23]. The 41 chloroplast genomes analyzed here had highly conserved structure, with minor variation between species. Expansion and contraction events at SC/IR boundaries often give rise to variation in chloroplast genome length [27], but *Ilex* plastomes varied by at most 901 bp in length. Although we detected small variations around IR junctions, the IR regions of the *Ilex* chloroplast genomes examined showed only modest expansions or contractions; IR regions varied from 25,080 to 26,121 bp, while LSC regions varied by about 900 bp (Table 1).

Variation in intergenic spacer regions, as well as gene loss and gain, also play important roles in shaping plant chloroplast genomes [23, 28]. In the seven newly sequenced chloroplast genomes, except for *I. dasyphylla*, all species lacked the gene *psbI*. Plastid gene loss has been previously documented in *Ilex—*specifically, deletions in the *trnT-trnL* and *ycf4-cemA* spacers of *I. graciliflora* [29]—which suggests that gene loss may be a relatively more common force influencing *Ilex* plastome architecture.

### Repetitive sequence analysis

Chloroplast simple sequence repeats (SSRs) are commonly employed in population genetics and evolutionary studies because of their high rate of polymorphism and abundant variation at the species level [30]. We identified a total of 2146 SSR loci from the 41 *Ilex* chloroplast genomes. Few population genetic studies have used SSRs in *Ilex*, and these newly identified loci will facilitate future research into genetic diversity, structure, and phylogeography at the population, intraspecific, and cultivar levels in *Ilex*.

Long repeat sequences with lengths greater than 30 bp play important roles in creating insertion/deletion mismatches and rearrangements that lead to genomic variation [31–34]. We found that the number of long repeat sequences in *Ilex* is high compared to some other angiosperm clades (e.g., 364 long repeats in Oxalidaceae [35]; 403 in *Veratrum* [36]; 32 in *Oresitrophe rupifraga*, and 34 in *Mukdenia rossiiand* [37]). Among these long repeats, forward, palindromic, and tandem repeats were rather common, accounting for 33.84, 30.81, and 34.44% of the total number of repeats, respectively, while complementary and reverse repeats were quite rare, only accounting for 0.42 and 0.49%, respectively.

### Hypervariable regions

Hypervariable regions often provide a wealth of phylogenetic information and can be used to delimit closely related taxa [38, 39]. In general, IR regions are more highly conserved than SSC and LSC regions [40]. We identified eight hypervariable regions in *Ilex* plastomes, including four genes and four genes with flanking regions. Consistent with angiosperm-wide patterns of plastomes variability [32, 33], all hypervariable loci were distributed in the SC regions, while IR regions exhibited low variation.

To date, phylogenetic analyses of *Ilex* have been based on a handful of plastid markers (mainly *atpB-rbcL*, *psbA-trnH*, *rbcL*, and *trnL-trnF*), which could not resolve many interspecific relationships [1, 2, 10, 13, 15, 41–43]. When comparing these markers to the highly variable regions identified here, only one (*rbcL*) has been used to construct phylogenies. We believe that these eight highly variable regions will be useful for phylogenetic inference and DNA barcoding in *Ilex*. However, further studies are required to evaluate the strength of these regions for identifying and delimiting species.

### Phylogenetic inference

There have been numerous attempts to resolve relationships amongst major *Ilex* lineages and test the consistency between molecular phylogenetics and traditional taxonomic systems based on morphology evidence [10–15, 26, 41]. A dearth of genetic data has resulted in poor resolution at the species level and weak support at most nodes in the *Ilex* phylogeny [10, 12–14, 26, 41]. These limitations can be addressed by using longer and more variable DNA sequences [44], such as complete chloroplast genomes [16, 21, 29, 45].

We present a well resolved and highly supported phylogeny of *Ilex*, and—in combination with macro-morphological and distribution information—suggest five clades (A-E) that are not generally congruent with traditional taxonomic systems. Clades A-E were largely consistent with previous plastid phylogenies, but relationships among clades differed significantly [10, 13, 15]. Our results showed that the American groups (Clade E) and the Eurasia groups (Clade F) were sister, and together formed the earliest diverging *Ilex* lineage, sister to a large clade containing the mostly Asian Clades A–C. In contrast, Manen [13] found the American (Group 3) and Eurasia (Group 4) groups to be among the most recently diverged lineages. The discordance between these results likely stems from the choice of loci included in analyses; previous studies have generally used less variable regions that led to low resolution among major clades [10, 13].

Our results highlight inconsistencies between molecular phylogenetics and traditional taxonomic systems. Almost all traditionally recognized subgenera, sections, and series included in our analysis were paraphyletic (all but sect. *Aquifolium*). Although the resolution of earlier phylogenetic trees was quite low, they indicated significant cyto-nuclear discordance, with nuclear phylogenies generally more consistent with traditional morphological classifications [13]. We confirmed the incongruences between plastid data and morphological systems by improving the resolution of the plastid phylogeny using complete chloroplast genomes.

Species found in close geographic proximity are often assumed to be closely related. This is accurate for most of the *Ilex* species in our study, including *I. cornuta*, *I. dasyphylly*, *I. latifolia*, and *I. integra*. However, both *I. pubescens* and *I. lohfauensis* were non-monophyletic in our analysis: the two accessions of *I. pubescens* were placed in two distinct clades (A and B), while the two accessions of *I. lohfauensis* were paraphyletic with respect to *I. championii*. Three samples of *I. viridis* were placed with the morphologically similar species *I. trifloral*. Non-monophyletic species may result from chloroplast capture or hybridization events [13, 41, 43], or stem from misidentification. Further phylogenetic studies are needed to continue to clarify relationships and taxonomy in *Ilex*.

## Conclusions

We conducted comparative and phylogenetic analyses of 41 *Ilex* chloroplast genomes, including seven newly sequenced taxa. To reach a more complete understanding of the evolutionary history of the clade, future studies should focus on phylogenetic reconstructions based on nuclear DNA. We suggest using low-copy nuclear genes from genome-skimming data, which can provide better resolution than traditional, short nuclear DNA markers (e.g., ITS). Incorporating nuclear phylogenies with existing phylogenies based on complete chloroplast genomes, as well as morphology, with enhance our understanding of the complex evolutionary history of *Ilex*.

## Materials and methods

### Taxon sampling, DNA extraction, and sequencing

Seven species of *Ilex* (*I. dasyphylla*, *I. fukienensis*, *I. lohfauensis*, *I. venusta*, *I. viridis*, *I. yunnanensis*, *I. zhejiangensis Ilex fukienensis*, *I. venusta*, and *I. zhejiangensis*) were collected from their native ranges in China. Fresh leaf tissues were collected in the field and stored in silica gel prior to DNA extraction. Voucher specimens were prepared and deposited at the herbarium of Nanjing Forestry University (NF). In addition, 34 complete chloroplast genomes of *Ilex* species that are publicly available in NCBI GenBank were downloaded with annotations (Additional file 1: Table S4). Based on the classification of *Ilex* that is generally accepted [25], the current dataset comprised species from six sections and 11 series of the genus *Ilex*.

Total genomic DNA was extracted using the Plant Genomic DNA Kit (Tiangen Biotech, China) following the manufacturer’s protocol. DNA extractions were visualized on agarose gels and quantified using a Qubit 2.0 (Life Technologies) for integrity, purity, and concentration. The qualified DNA (≥50 ng) was used to construct a paired-end (2 × 150 bp) library, and sequencing was conducted on a HiSeq X Ten platform (Illumina, USA).

### Chloroplast genome assembly and annotation

Raw reads were filtered with fastp v.0.20.0 software [46] to remove low-quality reads. The filtered data were then fed into the NOVOPlasty 2.6.3 [47] pipeline for genome assembly, with the *rbcL* gene sequence of *I. latifolia* (Accession number: KX897017) as the seed sequence and the chloroplast genome sequence of *I. latifolia* (Accession number: MN688228) as reference genome. A contig was obtained at the end of the process, and annotation was conducted using Plann [48], in which the annotated chloroplast genome of *I. latifolia* (Accession number: MN688228) was set as reference. Start and stop codons in the chloroplast genomes were manually corrected using DOGMA [49], and tRNA genes were verified with tRNA scan-SE v2.0.3 within in GeSeq [50] using default parameters. Circular chloroplast genome maps were visualized using OrganellarGenomeDRAW [51].

### Comparative genomic analyses

Sequence alignment of the 41 complete chloroplast genomes was carried out using MAFFT v.7 [52] and the alignment was further trimmed using trimAI v1.2 using the “-gappyout” setting [53]. The expansions and contractions of IR regions were visualized using IRscope [54] online and then was manually checked. The nucleotide diversity (Pi) was estimated using DnaSP v.5 [55] with a step size of 200 bp and a window length of 800 bp. The genome variability across the 41 species of *Ilex* was assessed using mVISTA [56] in Shuffle-LAGAN mode. The Mauve version 2.3.1 [57] plug-in available in Geneious version 11.0.3 [58] was used to identify locally collinear blocks among the chloroplast genomes with default parameters.

### Repeat sequence identification

The number of large repeats, including forward, palindromic, reverse, and complementary repeats were identified using onlineREPuter [59] according to the following criteria: sequence identities of 90%, cutoff point at ≥30 bp, Hamming distance set at 3, and a minimum repeat size of 30 bp. Tandem Repeat Finder [60] was used to analyze tandem repeat sequences with the default parameters. SSRs were identified using web-MISA [61], with minimum repeat number set at 10, 5, 4, 3, 3, and 3 for mono-, di-, tri-, tetra-, penta-, and hexanucleotides, respectively. Compound SSRs were detected by identifying independent SSRs that were separated by less than 100 nucleotides and were combined into one.

### Phylogenetic analyses

Phylogenetic analyses were conducted using 52 complete chloroplast genomes and 75 protein-coding genes. A total of 39 *Ilex* species from six sections and 11 series were included in the phylogenetic analyses. Based Yao et al. [26], *Helwingia himalaica* (Accession number: NC031370) was used as the outgroup. Genome alignment was carried out using MAFFT v.7 [52] and then trimmed using trimAI v1.2 with the “-gappyout” setting [53].

Maximum likelihood (ML) analyses were conducted using IQ-tree [62] with 10,000 ultrafast bootstrap (UFBS) replicates [63]. According to Bayesian information criterion (BIC), the best fitting substitution models that were estimated using ModelFinder [64] were GTR + F + I + G4 for the complete chloroplast genome sequences and GY + F + R3 for the protein-coding genes, respectively. Bayesian inference (BI) analysis was carried out using MrBayes version 3.2 [65], as implemented in CIPRES [66]. The Markov chain Monte Carlo analysis was executed for 2,000,000,000 generations, with four chains (one cold and three heated), each starting with a random tree, and sampled at every 1000 generations. Convergence of runs was accepted when the average standard deviation (*d*) of split frequencies was < 0.01. The first 25% of the trees were discarded as burn-in, and the remaining trees were used to construct majority-rule consensus trees. The final trees from both analyses were visualized using FigTree v.1.4.2 [67].

## Supplementary Information

**Additional file 1.**

**Additional file 2: Figure S1.** Sequence alignment of 41 *Ilex* chloroplast genomes using mVISTA with *I. szechwanensis* as a reference. The vertical scale indicates the percent identity, ranging from 50% to 100%. The horizontal axis shows the location within the plastomes. Genome regions are color-coded as exon, intron, and untranslated regions (UTRs). **Figure S2.** Mauve multiple alignment of 41 *Ilex* chloroplast genomes revealing no interspecific rearrangements. **Figure S3.** Phylogenetic trees inferred from maximum likelihood (ML) and Bayesian inference (BI) analyses based on 75 protein-coding genes. Numbers near the nodes are ML bootstrap support values (BS, left of the slashes) and Bayesian posterior probabilities (PP, right of the slashes). 100% BS or 1.00 PP are indicated by asterisks. Incongruences between the BI and ML trees are indicated by dashes. Recognized groups (major clades) were also marked by the right-hand black bar.

## Data Availability

All data generated or analyzed in this study were included in this published article and the Additional files. The complete chloroplast genomes of the seven newly sequenced *Ilex* species were submitted to GenBank and the accession numbers can be found in Additional file 1: Table S4. All raw reads are available in the short sequence archive under accession no. PRJNA768933. All complete genome sequences used in this study were downloaded from NCBI (https://www.ncbi.nlm.nih.gov), and the accession numbers can be found in Additional file 1: Table S4.
