# Supplementary figures and images for: Comparative analysis of complete Ilex (Aquifoliaceae) chloroplast genomes: insights into evolutionary dynamics and phylogenetic relationships
Source: BMC Genomics. 2022 Mar 14;23:203. doi: 10.1186/s12864-022-08397-9 (PMC8922745; doi:10.1186/s12864-022-08397-9)

[illegible]

Figure S2

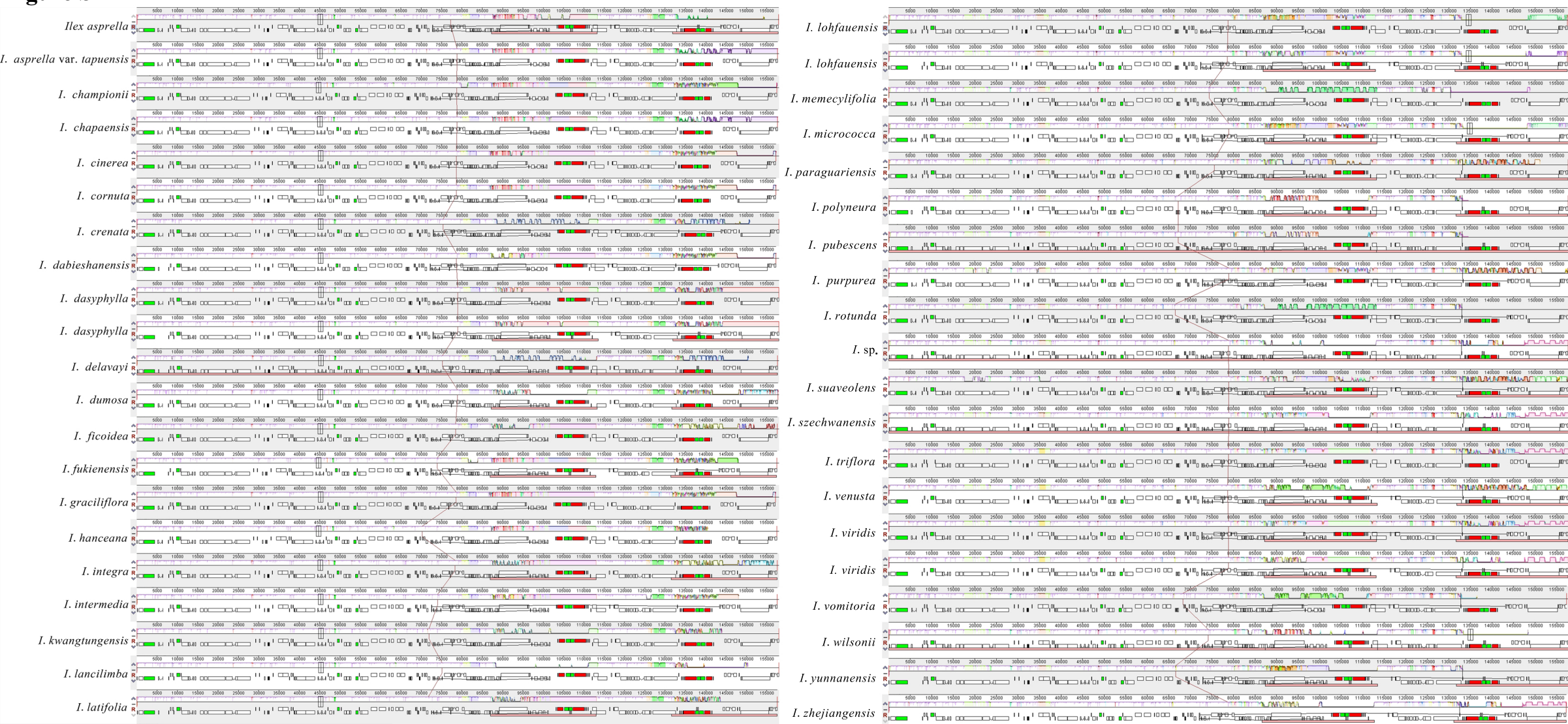

**Figure S3**

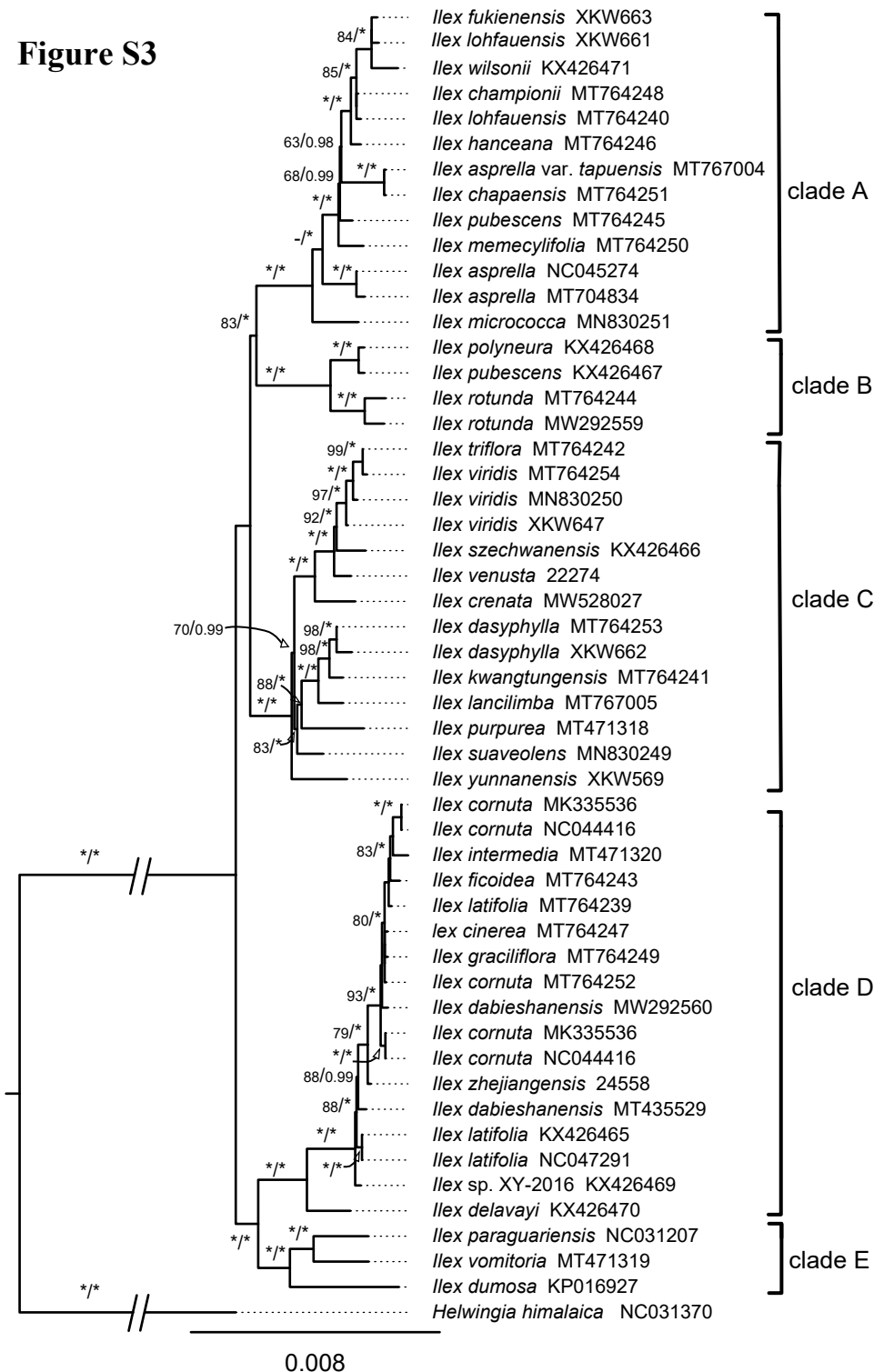

Supplement: Supplementary file 2 — Additional file 2: Figure S1. Sequence alignment of 41 Ilex chloroplast genomes using mVISTA with I. szechwanensis as a reference. The vertical scale indicates the percent identity, ranging from 50% to 100%. The horizontal axis shows the location within the plastomes. Genome regions are color-coded as exon, intron, and untranslated regions (UTRs). Figure S2. Mauve multiple alignment of 41 Ilex chloroplast genomes revealing no interspecific rearrangements. Figure S3. Phylogenetic trees inferred from maximum likelihood (ML) and Bayesian inference (BI) analyses based on 75 protein-coding genes. Numbers near the nodes are ML bootstrap support values (BS, left of the slashes) and Bayesian posterior probabilities (PP, right of the slashes). 100% BS or 1.00 PP are indicated by asterisks. Incongruences between the BI and ML trees are indicated by dashes. Recognized groups (major clades) were also marked by the right-hand black bar. [file 12864_2022_8397_MOESM2_ESM.pdf]
